# PReS-FINAL-2349: Spectrum of thrombotic and non-thrombotic manifestations in 159 children with positive antiphospholipid antibodies

**DOI:** 10.1186/1546-0096-11-S2-P339

**Published:** 2013-12-05

**Authors:** M Rozic, A Trampus-Bakija, Z Rener-Primec, L Kitanovski, T Kveder, T Avcin

**Affiliations:** 1 Department of Infectious Diseases, Ljubljana, Slovenia; 2 Children's Hospital, Ljubljana, Slovenia; 3 Department of Rheumatology, University Medical Center Ljubljana, Ljubljana, Slovenia

## Introduction

Antiphospholipid antibodies (aPL) play the central pathogenic role in antiphospholipid syndrome (APS) characterized by arterial and venous thrombosis, recurrent fetal loss and persistent circulating aPL. The diagnostic criteria of definite APS are not entirely applicable in pediatric population. An international multicentric project named Ped-APS registry included 121 patients with APS onset before 18th birthday. Almost half of them (49.5%) had an associated autoimmune disease. In this study a large percentage of children with aPL-related thrombotic event had at the time of the initial thrombotic event associated nonthrombotic clinical manifestations including: hematological manifestations (38%), dermatological manifestations (18%) and nonthrombotic neurological manifestations (16%).

## Objectives

To evaluate the spectrum of thrombotic and non-thrombotic clinical manifestations associated with aPL, namely anticardiolipin antibodies (aCL), anti-ß_2_-glycoprotein I antibodies (anti-ß_2_GPI) and lupus anticoagulant (LA), among children with positive laboratory results when tested for the presence of aPL.

## Methods

Pediatric patients in a tertiary care hospital were according to the clinical judgment of the treating physician tested for the presence of aPL and the laboratory results were saved in a computer database. In this single-center bidirectional study we included 159 consecutive patients from the database that tested medium or highly positive for aCL and/or positive for anti-ß_2_GPI and/or positive for LA at least once in the period from January 1997 to July 2012. Clinical manifestations of these patients were then evaluated, specifically thrombosis, nonthrombotic neurological and hematological manifestations, skin disorders and cardiac valve disease.

## Results

Of all 159 patients (61 boys and 98 girls, mean age at the occurrence of symptoms was 11.4 years, range 1 to 18 years of age) 55 had an underlying systemic disease (Systemic lupus erythematosus 31, Juvenile idiopathic arthritis 19, Scleroderma 2, Primary Raynaud's syndrome 2 and Sarcoidosis 1) and 8 had other autoimmune disease. Sixty-six out of 159 (42%) patients presented with one aPL-related clinical manifestation, 18 (11%) with two and 5 (3%) patients presented with three or more aPL related clinical manifestations. Of the aPL related clinical manifestations thromboses occurred in 25 patients (venous 16, arterial thrombosis 9, recurrent thrombosis 2), nonthrombotic neurological disorders were present in 25 patients (seizures 11, migraine 7, chorea 2, other 5), hematological disorders in 48 (thrombocytopenia 24, autoimmune hemolytic anemia 10, leucopenia 9, Evans syndrome 5), skin disorders in 19 (Raynaud's phenomenon 11, livedo reticularis 8) and a cardiac valve disease in 5.

## Conclusion

In an unselected group of children with positive aPL nonthrombotic clinical manifestations were more frequent than thrombotic events. The most common clinical manifestations associated with aPL in children are thrombocytopenia, followed by venous thrombosis, seizures and Raynaud's phenomenon.

## Disclosure of interest

None declared.

